# Changes in Aggressiveness of the *Ascochyta lentis* Population in Southern Australia

**DOI:** 10.3389/fpls.2016.00393

**Published:** 2016-03-31

**Authors:** Jennifer Davidson, Gabriel Smetham, Michelle H. Russ, Larn McMurray, Matthew Rodda, Marzena Krysinska-Kaczmarek, Rebecca Ford

**Affiliations:** ^1^Pulse and Oilseed Pathology, Plant Health and Biosecurity, Sustainable Systems, South Australian Research and Development InstituteAdelaide, SA, Australia; ^2^Faculty of Veterinary and Agricultural Sciences, University of MelbourneParkville, VIC, Australia; ^3^New Variety Agronomy, Sustainable Systems, South Australian Research and Development InstituteClare, SA, Australia; ^4^Biosciences Research, Department of Economic Development, Jobs, Transport and ResourcesHorsham, VIC, Australia; ^5^School of Natural Sciences, Environmental Futures Research Institute, Griffith UniversityNathan, QLD, Australia

**Keywords:** ascochyta blight, *Didymella lentis*, lentil, *Lens culinaris*, aggressiveness

## Abstract

Anecdotal evidence identified a change in the reaction of the resistant lentil cv Nipper to ascochyta blight in South Australia in 2010 and subsequent seasons, leading to infection. This study investigated field reactions of lentil cultivars against *Ascochyta lentis* and the pathogenic variability of the *A. lentis* population in southern Australia on commonly grown cultivars and on parental germplasm used in the Australian lentil breeding program. Disease data recorded in agronomic and plant breeder field trials from 2005 to 2014 in southern Australia confirmed the change in reaction on the foliage of the previously resistant cvs Nipper and Northfield. Cultivar responses to seed staining from *A. lentis* did not change. The change in foliar response was confirmed in a series of controlled environment experiments using single, conidium-derived, isolates of *A. lentis* collected over different years and inoculated onto differential host sets. Specific isolate/cultivar interactions produced a significant range of disease reactions from high to low aggressiveness with a greater percentage of isolates more aggressive on cvs Nipper, Northfield and PBA Flash than previously detected. Specific isolates were tested against Australian lentil cultivars and breeding lines in controlled conditions, again verifying the aggressiveness on cv Nipper. A small percentage of isolates collected prior to the commercial release of cv Nipper were also able to infect this cultivar indicating a natural variability of the *A. lentis* population which subsequently may have been selected in response to high cropping intensity of cv Nipper. Spore release studies from naturally infested lentil stubbles collected from commercial crops also resulted in a high percentage of infection on the previously resistant cvs Nipper and Northfield. Less than 10% of the lesions developed on the resistant differentials ILL7537 and cv Indianhead. Pathogenic variation within the seasonal populations was not affected by the cultivar from which the stubble was sourced, further indicating a natural variability in aggressiveness. The impact of dominant cultivars in cropping systems and loss of effective disease resistance is discussed. Future studies are needed to determine if levels of aggressiveness among *A. lentis* isolates are increasing against a range of elite cultivars.

## Introduction

*Ascochyta lentis* (teleomorph *Didymella lentis*) is the causal agent of ascochyta blight of lentil (*Lens culinaris*) (Kaiser et al., 1997), a disease of global importance and considered the major biotic constraint to lentil production in Australia (Salam et al., 2011). Australia is currently the second largest exporter of lentil behind Canada (FAOSTAT, 2014), producing 348,000 tons in 2014. Production is almost entirely in winter cropping areas of Victoria and South Australia, with less than 1% production in the states of New South Wales and Western Australia (Pulse Australia, 2014). On average 3% of arable land is cropped to lentils across South Australia each year and less than 2% of arable land across Victoria (Pulse Australia, 2014). Seasons characterized by frequent and prolonged winter rainfall events as can occur in these regions favor *A. lentis* infection and development of ascochyta blight leading to yield losses and reduced marketability of resultant stained and distorted seeds (Hawthorne et al., 2012).

*A. lentis* is specific to cultivated and wild species of lentil (Tullu et al., 2010). It is morphologically indistinct from *A. fabae* but the latter is unable to infect lentil species (Kaiser et al., 1997). Movement of the host germplasm has disseminated the pathogen worldwide (Kaiser, 1997) where it is primarily introduced to new sites through infected seed (Morrall and Sheppard, 1981; Kaiser and Hannan, 1986; Nasir and Bretag, 1997b). Wind dispersal of ascospores from infected lentil stubble into neighboring fields is considered the primary source of inoculum in Australia (Hawthorne et al., 2012) while splash dispersal of asexual pycnidiospores in prolonged damp conditions leads to epidemics. Sexual ascospores are produced on lentil stubble from the previous crop when both fungal mating types, MAT1-1 and MAT1-2, are present (Kaiser and Hellier, 1993), leading to increased genetic diversity and adaptive potential (Martin et al., 2013). Both mating types are present in Australia (Skiba and Pang, 2003) and the teleomorph has been identified in the field in both Victoria and Western Australia (Galloway et al., 2004). In Australia the ascospores are produced during the growing season in late autumn and winter (May to July) and are wind dispersed to a distance of 50 m from infected stubble (Galloway and MacLeod, 2002).

Control of the disease currently consists of the integrated selection of the most resistant varieties and best cultural practices, plus applications of fungicides on seed and foliage (Hawthorne et al., 2012). Fungicide applications are a considerable cost, both financially and environmentally, and can be difficult to apply in a timely fashion due to adverse weather and soil conditions therefore the development of highly resistant lentil varieties continues to be a primary breeding goal. Traditional breeding techniques have been used to date since the sources of genetic resistance to *A. lentis* are still largely uncharacterised (Ahmad et al., 1997; Ford et al., 1999; Gupta et al., 2012).

The cv Northfield, a selection from the ICARDA breeding line ILL5588 (originally from Jordan) was identified as resistant to ascochyta blight (Ali, 1995), and registered in 1995 to become one of the first cultivars to be grown in Australia, particularly in South Australia (Muehlbauer et al., 2009). Subsequently, it was replaced by the cv Nugget with moderate resistance to ascochyta blight (Hawthorne et al., 2011) and by cv Nipper with high resistance (McMurray et al., 2011). The cv Nipper, the progeny of two resistant cultivars *viz*. Indianhead and Northfield (Pulse Australia, 2011), was released to industry in 2006 (Taylor et al., 2007). Like Northfield, the parental line Indianhead also has a high level of resistance to ascochyta blight (Ye et al., 2001) and has been used extensively in the Australian lentil breeding program, along with the resistant breeding line ILL7537.

Resistant hosts, however, may instigate the selection of more aggressive individuals (Pariaud et al., 2009), where aggressiveness is “the quantitative variation of pathogenicity on susceptible hosts.” An early RAPD study on the *A. lentis* population in Australia (Ford et al., 2000) found the diversity of this fungal population was similar to that of isolates originating from outside of Australia. The authors concluded that the diversity came about through multiple introductions from different international sources and warned that this diversity and the presence of mating types provided a high potential for adaptation via sexual reproduction. Glasshouse studies of aggressiveness of *A. lentis* isolates in Australia in the late 1990's (Nasir and Bretag, 1997a, 1998), described 39 isolates as five or six pathotypes. Similarly, the other published Australian study to date (Sambasivam, 2011) also classified 17 isolates into six pathotypes although on a different host set, making comparisons difficult. This range of reactions from mostly resistant to highly susceptible is consistent with international studies (Bayaa et al., 1994; Ahmed and Morrall, 1996; Ahmed et al., 1996). However, a Canadian study of a larger number of isolates (84) against 10 lentil differentials indicated there was a continuum of aggressiveness without cultivar specificity (Ahmed et al., 1996).

Anecdotal evidence identified of a change in reaction to ascochyta blight on the cv Nipper in South Australia in 2010 and subsequent seasons, leading to infection on this cultivar. This study investigates the pathogenic variability of the *A. lentis* population in southern Australia on commonly grown cultivars and on parental germplasm used in the Australian lentil breeding program. A suite of lentil field trials are conducted each season across southern Australia for agronomic and breeding purposes and these trials were used, along with commercial crops, as a resource for determining host reaction to natural pathogen infection and for pathogen collection. Therefore, the aims of this study were to determine (1) The field reactions of lentil hosts against *A. lentis* over a number of seasons (2) The overall range of aggressiveness among recent Australian isolates of *A. lentis* against lentil differentials under a controlled environment, (3) If isolates with higher aggressiveness than identified from previous studies are present, and (4) If distinct isolate per host interactions exist in the Australian population.

## Materials and methods

### Field trials

Ascochyta blight naturally infected a number of lentil field trials, including Pulse Breeding Australia (PBA) selection trials, National Variety Trials (NVT; http://www.nvtonline.com.au/) and agronomic research trials in South Australia in 2005, 2010, 2013 and Victoria in 2014. These trials were assessed for disease as described below to provide data to breeders and agronomists on cultivar reactions and efficacy of disease management practices. In these seasons, rainfall was up to 189% above the 50 year long term average (105 mm compared to 90 mm long term average) (Bureau of Meteorology, 2016) in August and September when crops are starting to flower and conidial splash of *A. lentis* spreads the pathogen. Very limited ascochyta blight was evident in the intervening years due to dry seasonal conditions (18–84 mm during August and September) which prevented the development and spread of disease. Trials (Table 1) were randomized blocks, sown in 6.75 or 13.5 m^2^ plots, with 3 replicates, and trial management represented local grower practice in the region with respect to sowing date, seeding rate, fertilizer, herbicides, and pesticides. All seed was treated with P-Pickle T® (a.i. 360gL^−1^ thiram plus 200 gL^−1^ thiabendazole) fungicide seed dressing at 200 ml per 100 kg of seed prior to sowing.

**Table 1:** **Ascochyta blight scores on foliage of lentil lines in field trials in South Australia and Victoria from 2005 to 2014**.

**Site**		**Melton**	**Paskeville Trial 1**	**Paskeville Trial 2**	**Melton Trial 1**	**Melton Trial 2**	**Maitland**	**Maitland**	**Minlaton**	**Willamulka**	**Mallala**	**Horsham**
**Year**		**2005**	**2010**	**2010**	**2010**	**2010**	**2010**	**2013**	**2013**	**2013**	**2013**	**2014**
**Disease scores**		**1–9**^a^	**1–9**	**1–9**	**1–9**	**1–9**	**1–9**	**%LAD**^b^	**%LAD**	**%LAD**	**%LAD**	**Sqrt %LAD**
**Lentil cultivar**	**Foliar resistance rating**											
Aldinga	MR/MS^c^				3.0	3.2					15.0	
Boomer	MR	5.6	2.5	1.7	1.0	1.3	1.0				0.0	1.1 (1.2)^d^
Cumra	S				5.3							
Nipper	R-MR/MS^e^	1.0	3.8	5.1	3.0	2.7	4.3	6.7	5.0	3.7	13.3	2.2 (4.9)
Northfield	R-MR/MS	1.0	2.5		3.7	1.8					3.3	1.9 (3.5)
Nugget	MR/MS	6.0	4.3	6.1	4.0	4.2	4.3	6.7	8.3	4.0	8.3	2.2 (5.2)
PBA Ace	R						1.2	0.0	0.0	0.0	0.0	0.4 (0.1)
PBA Blitz	MR						2.3	1.3	0.7	0.0	3.3	1.2 (1.4)
PBA Bounty	MR/MS		3.5	4.5	3.3	2.8	3.7				10.0	2.1 (4.3)
PBA Flash	MS		6.3	5.4	4.0	3.5	6.7	8.3	11.7	9.3	28.3	3.5 (12.3)
PBA Herald XT	R				1.0	1.0	1.0	0.0	0.0	0.0	0.0	1.0 (1.0)
PBA Hurricane XT	MR							0.2	1.7	0.0	0.0	0.8 (0.4)
PBA Jumbo	MR/MS						1.7	4.0	5.0	0.3	8.3	2.3 (5.0)
Friedman's statistic (*P*-value)		25.74 (0.001)	25.0 (0.002)	45.2 (0.001)	32.1 (0.001)	36.3 (0.001)	44.0 (0.001)					
LSD (*P* ≤ 0.05)								2.4	2.2	1.3	5.7	0.3

a *Foliar score 1–9; 1, no disease; 3, individual leaf lesions; 5, leaf and stem lesions; 7, leaf, stem and pod lesions; 9, plant death*.

b *%Leaf Area Diseased (%LAD)*.

c *Cultivar foliar resistance rating designated by Pulse Breeding Australia; R, resistant; MR, moderately resistant; MR/MS, moderately resistant/moderately susceptible; MS, moderately susceptible; S, susceptible*.

d *Raw data in parentheses*.

e *Cultivars had a different field reaction over several seasons*.

In 2005 and 2010 ascochyta blight symptoms were scored on the foliage in each plot during flowering and podding growth stages (August to September), using the 1–9 categorical scale; 1 = no disease, 3 = individual leaf lesions, 5 = leaf and stem lesions, 7 = leaf, stem, and pod lesions, 9 = plant death. Data were analyzed with Friedman's non-parametric analysis of variance. In 2013 and 2014, the disease on foliage was assessed as % Leaf Area Diseased (%LAD) of total foliage in each plot during flowering or early podding and these data were analyzed using Analysis of Variance for randomized blocks in Genstat® version 16.

All trials were harvested at maturity and 100 seeds per plot were sampled at random from trials in 2005 and 2013. Ascochyta blight seed staining was scored on 2005 grain samples using a categorical scale of 0–3; 0 = no staining; 1 = ascochyta blight lesions ≤ 1 mm diameter; 2 = ascochyta blight lesions > 1 mm diameter and <25% seed coverage; 3 = ≥ 25% seed coverage. Seed from the 2013 trial at Mallala was scored using a 0–5 categorical scale; 0 = no staining; 1 = ascochyta blight lesions ≤ 1 mm diameter; 2 = ascochyta blight lesions > 1 mm and < 10% seed coverage; 3 = ascochyta blight lesions > 2 mm and < 10% seed coverage; 4 = ascochyta blight lesions > 3 mm and between 10–25% seed coverage; 5 = ≥ 25% seed coverage. The number of seeds in each category was summed and a disease index (DI) was calculated for each plot as follows:

$DI=\;[\sum_{n}^{i=0}{\left(d_{i}*s_{i}/N\right]}^{*}$ 100/C where; s_i_ refers to the number of seeds in each disease category, *d*_*i*_ is the value of the disease category, N is the total number of assessed seeds per plot and C is the number of disease categories. Data from 2005 were square root transformed to normalize residuals and analysis of variance was performed on the transformed DI. Data from PBA Mallala 2013 did not require square root transformation for analysis. All data were analyzed using Genstat® version 16 and significant differences were based on 95% confidence intervals.

### Isolate collection from field trials and commercial crops

Lentil plants with typical ascochyta blight leaf or stem lesions as well as seeds with ascochyta blight lesions were collected from the above-mentioned trials and from commercial crops in South Australia from 2010 to 2014, including the years with limited disease incidence, and from plant material in field trials in Victoria in 2012. Diseased plants were collected in August and September each year during the growing seasons and seeds were collected after harvest. The host cultivar and location was recorded for each collection. Diseased plant material was surface sterilized by dipping in 70% ethanol, followed by 30 s in 1% hypochlorite then rinsed in sterile water. Seeds were soaked in 2% hypochlorite for 2 min then drained through muslin cloth and dried on Whatman® sterile filter paper in a laminar flow. Seed or plant material were placed onto potato dextrose agar (PDA) (Oxoid®) amended with 0.01% streptomycin and plates were incubated 10–14 days under fluorescent lights (two Phillips TLD 36W/840 daylight tubes and one NEC black fluorescent light) for 12 h day/night at 22°C. The resulting isolates were identified as *A. lentis* based on the morphological characteristics of the conidia and cultures (Morrall and Sheppard, 1981). Single conidium-derived isolates were prepared and stored in sterile water at 4°C. An additional 17 single conidium-derived isolates of *A. lentis* had been collected from within South Australia and stored as described above at the SARDI Pulse and Oilseed Pathology Laboratory between 1989 and 2006, prior to the commercial release of cv Nipper.

#### Isolate collection from infested lentil stubble

After harvest in December 2012, lentil stubble naturally infested with *A. lentis* was collected from three commercial crops including two crops of cv PBA Flash (moderately susceptible to ascochyta blight) (Hawthorne et al., 2012) and one crop of cv Nipper. All crops were located within the Yorke Peninsula region of South Australia, which has a comparatively high intensity of lentil cropping (13% of arable land compared to the state average of 3% arable land) (Pulse Australia, 2014). The three stubbles were placed, separately, into large (150 × 75 cm) nylon mesh bags with 1 kg stubble approximately 10 cm deep in each bag. These were placed on benches, one bag per bench, in an external environment in a shadehouse at SARDI exposed to ambient conditions from 21st January 2013 to encourage release of spores.

Seed of eight lentil lines were sown, 25 pots per cultivar, four seedlings per pot (90 × 90 × 180 mm) filled with Van Schaik's Biogro (Biogro Pty. Ltd.) pine bark potting mix plus half a teaspoon of super fine agricultural lime (Biogro Pty. Ltd.) to raise pH to 7.0. These lentil lines were the ascochyta blight resistant sources in the PBA breeding program *viz*. cvs Northfield, Indianhead, breeding line ILL7537, and selected commercial cultivars grown in South Australia *viz*. cvs Nipper, PBA Flash, Nugget, PBA Herald XT (the latter resistant to ascochyta blight) (Hawthorne et al., 2012) as well as the susceptible cv Cumra (Siddique, 2000). On 25th June five pots of each cultivar containing 4 week old seedlings were placed around each bag of stubble; all pots were at equidistance and immediately adjacent to the stubble. Seedlings were watered as required. Following initial *A. lentis* symptom observation the infected leaves were detached each week to count and collect the lesions until 28th August 2013. Single conidium-derived isolates were produced from lesions, as described above.

This experiment was repeated the following year with lentil stubble naturally infested with ascochyta blight, collected after harvest in December 2013 from three commercial crops, *viz*. one crop of cv PBA Flash and two crops of cv Nipper, and stubble from a lentil trial consisting of a mixture of cvs Nipper and Northfield. One of the cv Nipper crops was in the lower north region of South Australia, where the density of lentil cropping is 4.5% of the arable land (PIRSA, 2014) while the other stubble lots were sourced from the Yorke Peninsula region. Stubble was incubated as described above from 10th December 2013 and pots of the lentil lines with 4 week old seedlings were sown on 6th May 2014 and placed adjacent to the stubble in ambient conditions as described above. Lesions of ascochyta blight were first observed on plants on 4th June, and infected leaves were detached each week to count and collect the lesions until 30th July 2014. Single conidium-derived isolates were collected from these lesions and stored as described above.

The cumulative number of lesions per pot were tested for homogeneity using Bartlett's variance homogeneity test and pooled data were analyzed using a generalized linear mixed model in Genstat® version 16. Where the homogeneity test was significant, data sets were analyzed separately. Significant differences were based on 95% confidence interval.

### Phenotyping isolates under controlled environment conditions

Single conidium-derived isolates were tested on differential sets of lentil lines in a series of four experiments at SARDI Pulse and Oilseed laboratory and three experiments at The University of Melbourne, Faculty of Veterinary and Agricultural Science. A fifth experiment was also conducted at SARDI comprising NVT lentil entries from 2014.

#### SARDI isolate phenotyping experiments

Seventeen single conidium-derived isolates collected between 1989 and 2006 (designated 1989–2006 collection), 63 isolates collected between 2010 and 2013 (designated 2010–2013 collection) and 22 isolates collected in 2014 (designated 2014 collection) were tested on a differential set of five lentil lines in a series of four experiments in controlled conditions. Two reference isolates from Victoria, AL4 from 1998 and Kewell from 2001 (Nasir and Bretag, 1997a; Nguyen et al., 2001), were also included in each experiment to enable the ranking of isolate aggressiveness across trials. The differential set comprised Indianhead, ILL7537, cv Northfield, cv Nipper and the susceptible cv Cumra. Disease ratings of these lines had been determined by field assessments in previous seasons and in growth room experimental conditions (Sambasivam, 2011).

Seed of the five lentil lines were sown separately into pots as described above. In three experiments, each comprising 21 different isolates collected from 2010 to 2013 as described above plus the two control isolates, each lentil line was sown into 96 pots, four seeds per pot, which were thinned to three seedlings per pot after emergence. A fourth experiment, comprising the 17 isolates from 1989 to 2006 plus the two control isolates, consisted of 80 pots per line while a fifth experiment, comprising the 22 isolates from 2014 and control isolates, consisted of 100 pots per line. The experiments were of a split plot design, with isolates as the main plots and lentil lines randomly allocated to subplots. After sowing, the pots were placed in a controlled environment room (CER) at 15°C, 12 h/ 12 h light/ dark cycle in 4 plastic tents (160 × 80 × 80 cm), one replicate per tent. Pots were watered by hand as required. Seedlings were inoculated after 2 weeks as described below.

Cultures of the isolates were grown for 14 d on PDA as previously described. A conidial suspension of each isolate was prepared by flooding the plates with sterile distilled water and gently rubbing the culture surface with a sterile glass rod to suspend the conidia. The spore concentration was determined by haemocytometer and adjusted to between 9 × 10^5^ to 1 × 10^6^ conidia per mL. Conidial suspensions of 75 mL per isolate were produced and surfactant Tween 20 (0.01%) (Merck Pty. Ltd.) was added. Each conidial suspension was sprayed separately until runoff onto four replicate pots of each lentil line. Control seedlings (four pots per lentil line) were sprayed with sterile distilled water plus Tween 20 (0.01%) until runoff.

After inoculation, an ultrasonic humidifier using reverse osmosis water was turned on in each tent for 2 h and then for 1 h each day until disease assessment to maintain leaf wetness. Disease was assessed on each seedling 10–14 days after inoculation as % area of plant diseased (%APD), incorporating leaf and stem lesions of the 4 nodes and internodes that were spray inoculated. Data were square root transformed to normalize residuals where necessary and each experiment was analyzed separately using split plot analysis of variance with isolate as the main plot in GenStat® version 16. Cultivar x isolate reactions were placed into category Resistant (0–4.2%APD), Moderately Resistant (> 4.2–8.5%APD), Moderately Susceptible (> 8.5–13.0%APD) or Susceptible (>13.0%APD) based on the least significant difference between mean disease scores. Chi-square analysis in GenStat® version 16 was used to compare the number of isolates within each resistant category per host for the isolates in the different collection periods and for cultivar of origin for the isolates. A comparison of disease severity scores was made between isolates collected before 2006 and the isolates collected after 2006. The data were first averaged within pots to reduce variability and to satisfy assumptions of normality. Data were logarithm transformed to stabilize variance except for among the hosts and a generalized mixed linear model in GenStat® version 16 was used to analyse the data for each host independently. Significant differences between the two collection periods were based on 95% confidence interval.

#### University of melbourne isolate phenotyping experiments

A total of 29 Australian *A. lentis* isolates were assessed (Table 2) in this experiment. These were predominately isolated in South Australia from field plants or seed stocks in 2010, 2011, and 2012. The other isolates were from field plants from Victoria isolated in 2012 with the exception of the two reference isolates Kewell and AL4. Most isolates were from lentil cvs PBA Flash and Nipper, three were from cv Nugget, two from cv Northfield and one from cv Aldinga. Inoculum was prepared as described above and the concentration adjusted to 10^6^ spores per ml before adding a drop of Tween 80 (Merck Pty. Ltd.). Control seedlings were sprayed with sterile water plus Tween 80. Plants were inoculated until runoff using a 500 ml hand sprayer producing a fine mist, and the pots rotated during the procedure to achieve an even spread of inoculum. The host differential set consisted of ILL7537, cvs Northfield, Indianhead, Nipper, PBA Flash and the susceptible check ILL6002. Each accession was sown as five seeds per 5 cm forestry tube filled with a 1:1 pine bark/sand mix, ameliorated with dolomite to achieve pH 7.0, and grown in a growth room at 20°C with a 12 h photoperiod. After 2 weeks, these were thinned to three seedlings per pot immediately prior to inoculation. Seedlings were watered to field capacity twice a week, and fertilized weekly from 2 weeks old with Nitrosol (Amgrow) as per the manufacturer's instructions.

**Table 2:** **Mean disease score at 28 days after inoculation for *Ascochyta lentis* isolate/cultivar interactions**.

**Cultivar**	**ILL7537**	**Indianhead**	**Nipper**	**Northfield**	**Flash**	**ILL6002**
**Foliar resistance rating**^a^	**R**	**R**	**R-MR/MS**^b^	**R-MR/MS**	**MS**	**S**
**ISOLATE**						
ALP2	3.40	2.90	4.90	5.74	6.74	7.24
P3040	3.16	3.83	5.50	5.00	5.83	7.00
FT12022	3.74	3.07	5.07	5.24	6.07	7.07
AL4	3.02	4.52	4.97	4.47	5.47	7.36
P3046	2.73	3.73	5.06	4.39	5.73	7.73
61/10	3.12	3.95	4.62	5.29	5.12	7.12
P3012	2.40	4.40	4.57	4.90	5.57	7.40
P3026	3.25	3.25	4.58	5.41	5.25	7.41
ALK1	3.29	3.46	4.13	5.29	5.63	6.79
P3044	2.64	3.80	4.80	4.64	5.30	6.80
FT12023	3.21	3.05	5.21	4.71	5.05	6.71
FT12013	3.37	3.04	5.04	5.54	4.87	6.04
FT10001	2.97	3.80	4.14	4.64	5.14	7.14
MEL1	2.57	3.57	4.23	4.23	5.73	7.40
ALM8	3.18	3.02	3.35	5.68	5.35	7.02
P3047	2.81	3.15	3.65	4.81	5.48	7.31
MEL2	2.63	3.79	4.63	3.79	5.63	6.63
68/10	2.91	3.07	4.24	4.74	5.41	6.74
P3065	2.66	3.16	4.16	4.83	5.83	6.33
FT12025	3.16	3.49	4.16	4.16	4.99	6.66
FT12029	3.05	2.71	3.21	5.55	5.88	5.88
FT10017	2.92	3.26	4.09	3.59	5.42	6.76
58/10	2.41	3.24	4.24	3.74	5.74	6.58
ALR1	3.35	2.52	3.68	4.85	4.68	6.18
FT10012	2.63	2.96	3.96	3.46	4.96	7.13
FT10007	2.76	2.93	5.26	3.93	3.59	6.26
48/10	2.65	2.98	4.15	3.15	4.32	6.98
FT10016	2.68	2.52	3.35	3.52	4.35	6.35
Kewell	2.64	3.09	3.31	3.25	3.75	6.48
Mean	2.94	3.32	4.35	4.57	5.27	6.84
Standard deviation	0.33	0.49	0.62	0.75	0.66	0.45

*Lentil cultivars are presented in descending overall resistance from left to right and isolates are listed in descending order of overall aggressiveness. Scores: 0 = no disease to 9 = severe disease/plant death. LSD 5% = 0.54–1.09*.

a *Cultivar foliar resistance rating designated by Pulse Breeding Australia; R, resistant; MR, moderately resistant; MR/MS, moderately resistant/moderately susceptible; MS, moderately susceptible; S, susceptible*.

b *Cultivars had a different field reaction over several seasons*.

The 29 isolates were tested using three separate randomized, nested, complete block design trials. Each trial assessed three plants/accession/treatment and trials were repeated three times (total of four replicates). There were 12 treatments per trial consisting of nine unknown isolates, two positive control isolates (AL4 and Kewell) and one uninoculated control. The following method was adapted from those previously used (Nasir and Bretag, 1997a; Ford et al., 1999; Sambasivam, 2011) to promote infection and maintain conditions for disease development. The six pots, each containing a different accession, were placed randomly in a solid 2 L plastic container, assigned a treatment, inoculated as described above then placed randomly in one of two 200 L plastic crates to minimize air flow present in the growth room. The crate also contained water (2–4 cm depth) to maintain humidity. The crate was covered tightly with a lid and wrapped in black plastic for 48 h post inoculation to provide dark conditions with 100% humidity to promote infection. After removal of the coverings, the crates were misted three times a day and covered with damp hessian for 48 h each week to provide conditions conducive to disease. The growth room conditions were the same as those described above for seedling production.

Final disease assessment was made on whole plants 28 days after inoculation (dai) when discrimination of disease reaction between susceptible and resistant plants was distinct (Ford et al., 1999). One observation was made from each seedling. The subjective 1–9 disease index used by previous researchers (Nasir and Bretag, 1997a; Ford et al., 1999; Sambasivam, 2011) was modified by specifying a size limit of small lesions and percentage leaf drop. The scores were: 1 = no visible disease symptoms; 3 = leaf lesions only, chlorosis of affected leaves, < 10% leaf drop; 5 = leaf lesions, up to 25% leaf drop, stem flecks or lesions < 2 mm; 7 = leaf lesions, up to 50% leaf drop, stem lesions > 2 mm; 9 = leaf lesions, potential defoliation, stem girdling, potential plant death.

Statistical analysis was performed using GenStat® version 16. Data from all three trials were then pooled and analyzed using Linear Mixed Model analysis. The use of the same two controls in each trial provided a means of ranking isolates across trials. Data from control seedlings was excluded from all analyses to prevent bias since the scores were consistently 1. Means of disease score were calculated for isolates, cvs and the isolate /cv interaction using Least Square Difference (LSD) 5%. Interaction plots for each of the three trials were performed using Minitab 16 Statistical Software to provide a means of observing deviations from common patterns of interaction. Mean with 95% confidence limit was used to compare aggressiveness of isolates originally isolated from cv PBA Flash or cv Nipper. Mean scores were used to place isolate reactions on cultivars into categories of Resistant (score 1), Moderately Resistant (score 1.1–4.9), Moderately Susceptible (score ≥ 5–6.0) or Susceptible (score > 6.0) (Nasir and Bretag, 1998).

#### NVT lentil lines tested against *A. lentis* isolates in controlled conditions at SARDI

Twenty seven lentil lines from 2013 NVT trials (Table 3) were tested in controlled conditions against isolate F13013 (collected in 2013) and isolate F10002 (collected in 2010) which were aggressive and non-aggressive, respectively, to cv Nipper based on the controlled screening described above. Isolate Kewell (collected in 2001) was also included as a control. This four replicate split plot experiment was conducted and analyzed as described above for SARDI experiments. Mean disease scores for each line from the controlled screening were regressed against mean disease field scores from Mallala 2013 and Horsham 2014 using linear regression in GenStat® version 16. The disease scores against the three isolates and the field disease scores were used to place each lentil line into one of six response groups.

**Table 3:** **Ascochyta blight disease scores on National Variety Trial lentil entries for 2014 inoculated with three separate isolates of *Ascochyta lentis* in a controlled environment room, and in Mallala Pulse Breeding Australia (PBA) trial in 2013 and Horsham PBA trial 2014**.

**Lentil line**	**Pedigree**	**Controlled environment study % area of plant diseased, averaged for 4 replicates** × **4 plants**			**Field trials % leaf area diseased (%LAD), averaged for 3 replicates**		**Response group**
		**Control isolate**	**Non-aggressive isolate on cv Nipper**	**Aggressive isolate on cv Nipper**	**Mallala PBA trial 2013**	**Horsham PBA trial 2014**	
		**Kewell**	**FT10002**	**FT13013**	**%LAD**	**Sqrt %LAD**	
Flash	ILL7685/Nugget	3.7	4.6	22.9	28.3	3.51 (12.3)^a^	1: Most susceptible line in these tests, lacking major resistance genes and little partial resistance
^b^CIPAL1421	CIPAL0105-EMS03/CIPAL611//PBA Blitz	8.1	5.8	15.0	4.3	2.19 (4.8)	2: Lacking major resistance genes but displays partial resistance in field
PBA Jumbo	Aldinga/CDC Matador	1.4	3.3	14.2	8.3	2.25 (5.0)	3a: Susceptible to the new isolate (FT13013)and some susceptibility to older isolate (FT10002)
Nugget	Northfield/ILL5714	1.8	5.5	12.5	8.3	2.80 (5.2)	
CIPAL1405	PBA Blitz/CIPAL804//CIPAL611	1.4	10.1	16.7	2.1	1.82 (3.3)	
Northfield		0.8	3.6	17.9	3.3	1.88 (3.5)	
PBA Greenfield	CIPAL205/Boomer//PBA Flash	0.0	3.6	5.9	1.7	2.37 (5.6)	3b: Susceptible to the new isolate (FT13013) and resistant to older isolates
Nipper	Indianhead/Northfield//Northfield	0.0	0.1	8.3	13.3	2.20 (4.9)	
PBA Bounty	ILL6788/Nugget	0.1	1.6	9.6	10.0	2.08 (4.3)	
CIPAL1403	CIPAL405/CIPAL503//PBA Flash	0.0	0.3	6.7	2.3	2.09 (4.4)	
CIPAL1404	Nipper/CIPAL401//PBA Flash	0.1	1.3	6.8	3.1	1.68 (2.8)	
PBA Blitz	Cumra/Indianhead//Cassab	1.0	5.3	3.1	3.3	1.18 (1.4)	4a: Moderate resistance to new isolate (FT13013) but some susceptibility to older isolate (FT10002)
PBA Giant	PBA Flash/Boomer	1.1	1.3	1.6	3.3	2.00 (4.0)	4b: Moderate resistance to tested isolates but some susceptibility in field
CIPAL1302	96-047L-99R099/CIPAL204//CIPAL401	13.8	0.0	0.1	0.0	0.53 (0.3)	5a: Resistant to recent isolates F13013 and FT10002 but susceptible to the control isolate Kewell; all except CIPAL901 have CDC Matador in pedigree (96-047L being a Nugget/ CDC Matador cross)
CIPAL1303	96-047L-99R099/CIPAL204//CIPAL401	7.1	0.0	0.9	0.0	0.41 (0.2)	
CIPAL1422	PBA Herald XT/PBA Bolt	8.4	0.0	0.1	0.0	0.94 (0.9)	
CIPAL901	CIPAL501/CIPAL205//PBA Flash	6.3	0.0	0.0	0.0	0.55 (0.3)	
CIPAL1204	96-047L-99R099/CIPAL0204// CIPAL0401	2.7	0.0	0.4	0.0	0.54 (0.3)	
CIPAL1402	99-068L-2-02H042/ 02-325*03HS001	3.4	0.1	0.0	0.0	0.46 (0.2)	
PBA Hurricane XT	PBA Flash/96-047L*99R060M3	8.12	0.1	0.1	0.0	0.67 (0.4)	
Boomer	Digger/Palouse	0.0	0.1	1.4	0.0	1.10 (1.2)	5b: Resistant to the recent isolates (FT13013 and FT10002) and resistant to the control isolate Kewell; all except PBA Jumbo2 and Boomer have CDC Matador in pedigree
CIPAL1401	PBA Bolt/02-325*03HS001	1.8	0.0	0.1	0.0	0.75 (0.6)	
PBA Jumbo2	CIPAL205/Boomer//CIPAL401	0.8	0.0	0.0	0.0	0.03 (0.0)	
PBA Bolt	ILL7685/96-047L*99R060	0.7	0.0	0.0	1.7	0.53 (0.3)	
PBA Herald XT	96-047L*99R060-EMS02	0.1	0.0	0.0	0.0	1.00 (1.0)	
CIPAL1301	PBA Bolt/02-325*03HS001	0.0	0.0	0.1	0.0	0.36 (0.1)	
PBA Ace	CIPAL501/96-047L*99R099	0.0	0.0	0.0	0.0	0.36 (0.1)	
Least significant difference (*P* < 0.05)			3.9		1.9	0.34	

a *Raw data in parentheses*.

b *CIPAL refers to lentil crosses made by the Coordinated Improvement Program of Australian Lentils multiplied for potential release*.

## Results

### Ascochyta blight in field trials

In 2005 at the Melton field site, significant differences (*P* = 0.001) in ascochyta blight occurred such that no ascochyta blight was recorded on foliage of the resistant cv Nipper or on the resistant cv Northfield while the moderately resistant cvs Nugget and Boomer recorded disease scores ranging from 3.4 to 6.0 at the same site (Table 1). Five years later in 2010, cv Nipper recorded similar disease scores to cv Nugget (up to 5.1 and 6.1, respectively) and in 2013 cv Nipper again had a similar disease score to cv Nugget at the Mallala trial (13.3%LAD and 8.3%LAD, respectively, Least Significant Difference [LSD] = 5.7, *P* < 0.05). The resistant cvs PBA Ace, PBA Bolt, PBA Herald XT, and PBA Hurricane XT recorded a maximum disease score of 1.8 in 2010 and between 0 to 3.3%LAD in the 2013 trials. At Horsham in 2014, cv Nipper had a similar disease score to cv Nugget (4.9 and 5.2%LAD respectively, LSD = 0.3, *P* < 0.05) while the resistant cultivars listed above recorded significantly less disease than cv Nipper (0.1–1.0%LAD) (Table 1).

The highest seed staining DI (Table 4) was on cvs Boomer (18.0) and Nugget (14.0) in the Melton 2005 trial (LSD = 0.5, *P* < 0.05). The DI from the Sandilands 2005 trial were generally lower than from Melton but again cvs Boomer and Nugget had significantly higher DI than cv Nipper or cv Northfield. Very low DI was recorded on cvs Nipper (0.70) and Northfield (0.41) in both trials. In the Mallala 2014 trial, the highest seed DI was on cvs PBA Jumbo (7.9) and PBA Flash (4.9) (LSD = 1.6, *P* < 0.05). All other cultivars, including Nipper and Northfield, had a DI not significantly greater than zero.

**Table 4:** **Ascochyta blight seed staining scores, Disease Index (DI), on seed harvested from cultivars in three field trials in South Australia at Melton (2005), Sandilands (2005) and Mallala (2014)**.

**Site**	**Melton**	**Sandilands**	**Mallala**
**Year**	**2005**	**2005**	**2013**
**Disease rating**	**Sqrt DI**^a^	**Sqrt DI**^a^	**DI**^b^
**LENTIL CULTIVAR**			
Boomer	4.17 (18.0)^c^	1.91 (3.9)	0.7
Nipper	0.71 (0.65)	0.69 (0.5)	0.4
Northfield	0.72 (0.60)	0.52 (0.3)	0.07
Nugget	3.72 (14.0)	1.00 (1.05)	1.4
PBA Ace			0.0
PBA Blitz			1.3
PBA Bounty			0.6
PBA Flash			4.9
PBA Herald XT			0.07
PBA Hurricane XT			0.3
PBA Jumbo			0.1
LSD (*P* ≤ 0.05)	0.5	0.3	1.6

a Disease Index (DI) assessed using a categorical scale of 0–3 whereby 0, no staining; 1, ascochyta blight lesions ≤ 1 mm diameter; 2, ascochyta blight lesions > 1 mm diameter and < 25% seed coverage; 3, ≥25% seed coverage. $DI=\;[\sum_{i=0}^{n}\;{\left(d_{i}*s_{i}/N\right]}^{*}$ 100/C where s_i_ refers to the number of seeds in each disease category, d_i_ is the value of the disease category, N is the total number of assessed seeds per plot and C is the number of disease categories.

b *Disease Index assessed using a 0–5 categorical scale; 0, no staining; 1, ascochyta blight lesions ≤ 1 mm diameter; 2, ascochyta blight lesions > 1 mm and < 10% seed coverage; 3, ascochyta blight lesions > 2 mm and < 10% seed coverage; 4, ascochyta blight lesions > 3 mm and between 10 and 25% seed coverage; 5, ≥25% seed coverage*.

c *Raw data in parentheses*.

#### Isolate collection from infested lentil stubble

**Figure 1:**
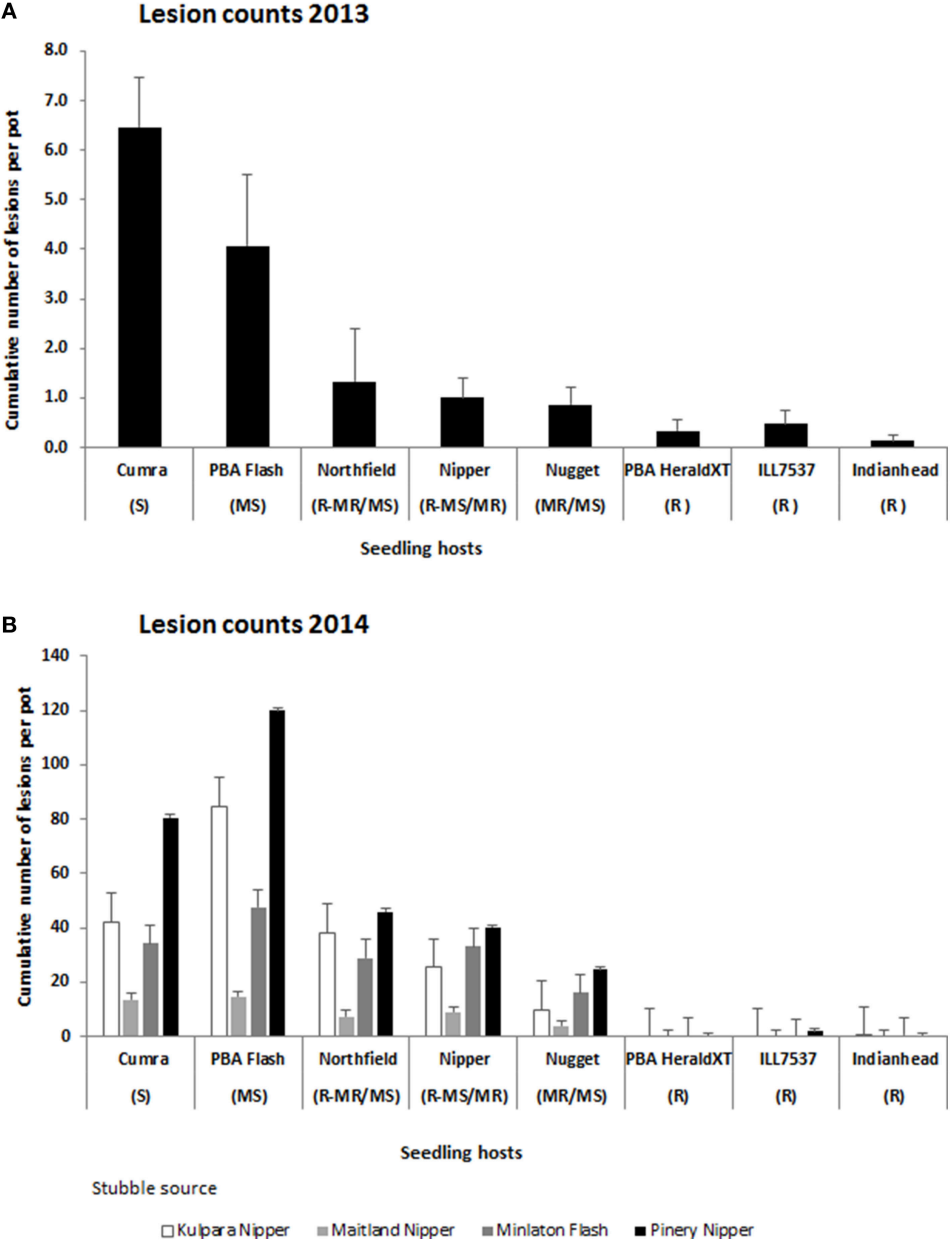
**Cumulative number of ascochyta blight lesions assessed on 5 lentil seedlings per pot adjacent to naturally infested lentil stubble from commercial crops or field trials incubated in (A) 2013, average of three stubble sets, LSD 5% = 2.1 and; (B) 2014, LSD 5% (interaction stubble set x seedling host) = 13.8**. Vertical bars represent standard errors of the means. R, resistant; MR, moderately resistant; MR/MS, moderately resistant/moderately susceptible; MS, moderately susceptible; S, susceptible.

Bartlett's variance homogeneity test was significant between years for the stubbles incubated in 2013 and 2014 (Chi-square 30.1 on 6 df, *P* < 0.001) but was not significant within each year, hence the 2013 and 2014 data sets were analyzed separately. In 2013 the origin of the stubble had no significant influence on lesion production but significant differences (*P* < 0.001) were observed in cultivar reactions such that the majority of the lesions developed on the susceptible cv Cumra and moderately susceptible cv PBA Flash, and least number of lesions developed on the remaining cultivars which ranged from an intermediate resistance (moderately resistant/moderately susceptible) to resistant (Figure 1A). In 2014 there was a significant interaction between stubble source and lesion host (*P* < 0.001). However responses mirrored those of 2013 in that for each stubble source the majority of lesions developed on either cv Cumra or cv PBA Flash, followed by either the cvs Nipper or Northfield and then cv Nugget. Least or no lesions developed on the three resistant cvs Indianhead, PBA Herald XT and ILL7537 (Figure 1B).

### Phenotyping isolates under controlled environment conditions

The interaction between cultivar and isolate was significantly different for disease scores in the SARDI and University of Melbourne tests. Disease scores in SARDI tests ranged from 0 to 33.2%APD with LSD_interaction_ (*P* < 0.05) ranging from 2.9 to 6.7 for individual experiments. The disease scores in the University of Melbourne tests ranged from 2.4 to 7.73 (1–9 scale) with LSD_interaction_ (*P* < 0.05) ranging from 0.54 to 1.09. The analyzed results were used to place the isolate reactions on the differential hosts into resistance and susceptible categories (Table 5). The majority of isolates in the SARDI collection (73–100%) caused susceptible or moderately susceptible reactions on cv Cumra, and all isolates screened at The University of Melbourne tests caused a susceptible reaction on ILL6002 (the most susceptible of the lines tested) while ILL7537 was the most resistant line in both series of tests (Sambasivam, 2011). The cv Indianhead was resistant or moderately resistant to all isolates in both SARDI and University of Melbourne tests, although a small amount of disease (<4.2%APD) was recorded on this line. The cv PBA Flash showed moderate disease reaction overall in line with its field rating (Hawthorne et al., 2012).

Table 5**Number of isolates from different collection periods that cause resistant or susceptible reactions when tested against differential hosts in controlled conditions at (A) SARDI and (B) University of Melbourne**.

**Table:** 

**Differential hosts**	**Isolate × Host resistance category**	***A. lentis*** **isolates**			**Chi-square between Collection 1 and 2 (*df* = 1)**	**Chi-square between Collection 1 and 3 (*df* = 1)**
		**Collection 1 1989-2006**	**2010–2013 Collection 2**	**Collection 3 2014**		
**(A) SARDI**						
Cumra^a^	Resistant^b^	0 (0%)	17 (27%)	1 (5%)	5.8 (*P* = 0.02)	Not significant
	Susceptible^c^	17 (100%)	46 (73%)	21 (95%)		
Northfield	Resistant	13 (77%)	50 (79%)	2 (9%)	0.07 (*P* = 0.08)	18.4 (P < 0.001)
	Susceptible	4 (23%)	13 (21%)	20 (91%)		
Nipper	Resistant	13 (77%)	55 (87%)	7 (32%)	Not significant	7.7 (P = 0.006)
	Susceptible	4 (23%)	8 (13%)	15 (68%)		
ILL7537	Resistant	17 (100%)	63 (100%)	22 (100%)	–^d^	–
	Susceptible	0 (0%)	0 (0%)	0 (0%)		
Indianhead	Resistant	17 (100%)	63 (100%)	22 (100%)	–	–
	Susceptible	0 (0%)	0 (0%)	0 (0%)		
	Total	17	63	22		

a *Susceptible check*.

b *Resistant (combined R and MR categories)* = ≤ *8.5% area of plant diseased*.

c *Susceptible (combined S and MS categories)* = > *8.5% area of plant diseased*.

d *Data could not be analyzed due to zeros in the Susceptible category*.

**Table:** 

**Differential hosts**	**Isolate** × **Host resistance category**	**Collection 4 2010–2013**	**Chi-square between Collections 2 and 4 (*****df*** = **1)**
**(B) UNIVERSITY OF MELBOURNE TESTS**			
ILL6002^a^	Resistant^b^	0 (0%)	
	Susceptible^c^	27 (100%)	
PBA Flash	Resistant	7 (26%)	
	Susceptible	20 (74%)	
Northfield	Resistant	18 (67%)	Not significant
	Susceptible	9 (33%)	
Nipper	Resistant	21 (78%)	Not significant
	Susceptible	6 (22%)	
ILL7537	Resistant	27 (100%)	–^d^
	Susceptible	0 (0%)	–
Indianhead	Resistant	27 (100%)	
	Susceptible	0 (0%)	
	Total	27	

a *Susceptible check*.

b *Resistant (combined R and MR categories) = 1–4.9 (1–9 scale)*.

c *Susceptible (combined S and MS categories) = ≥ 5.0 (1–9 scale)*.

d *Data could not be analyzed due to zeros in the S-MS category*.

In SARDI tests, the previously resistant cv Nipper was susceptible to a greater number of isolates in the more recent collection compared to earlier collections (Table 5). Only 23% of the isolates collected between 1989 and 2006 produced a susceptible or moderately susceptible reaction on this cultivar but this significantly increased (*P* = 0.006) to 68% in the 2014 isolate collection. The resistant cv Northfield was susceptible or moderately susceptible to 91% of isolates from the 2014 collection. This was an increase from 23% of isolates collected during 1989–2006 (*P* < 0.001). The percentage of isolates with a resistant reaction on the susceptible cv Cumra was significantly higher (*P* = 0.02) in the 2010–2013 collection compared to the 1989–2006 collection and the 2014 collection (Table 5). The severity of disease on cv Nipper assessed in the controlled conditions, averaged for isolates from each collection period, significantly increased (Wald statistic 15.4, *P* < 0.001) over time (Table 6); i.e., 4.0%APD by isolates collected from 1989 to 2006 compared to 6.5%APD for isolates collected after 2006. Similar results were obtained at The University of Melbourne whereby the cvs Northfield and Nipper appeared less resistant than in the previous study conducted by Sambasivam (2011). The disease severity on ILL7537 did not vary with the two collection periods in the SARDI tests while cv Indianhead had significantly less (Wald statistic 43.3, *P* < 0.001) severe reaction to the later isolates although disease scores were low (≤1%APD) on this host (Table 6).

**Table 6:** **Comparison of mean disease scores for *Ascochyta lentis* isolates collected prior to 2006 compared to isolates collected after 2006 tested on five lentil hosts in controlled conditions at SARDI**.

**Host (Foliar disease rating)**	**Mean of log (disease** +**0.05) (raw data in parentheses)**		**Standard error of difference**	**Wald statistic**	***P*-value**
	**1989–2006 collection**	**Post 2006 collection**			
Cumra (S)^a^	2.561 (14.1%)	2.312 (12.2%)	0.132	3.5	0.061
Northfield (R-MS/MR)^b^	1.52 (6.9%)	1.553 (7.3%)	0.185	0.0	1.0
Nipper (R-MS/MR)	0.815 (4.0%)	1.503 (6.5%)	0.176	15.4	8.75E-05
ILL7537 (R)	−0.571 (0.2%)	−0.5827 (0.1%)	0.048	0.1	0.752
Indianhead (R)	−0.155 (1.0%)	−0.6698 (0.01%)	0.0781	43.3	4.7E-11

a *Cultivar foliar disease rating designated by Pulse Breeding Australia; R, resistant, MR, moderately resistant; MR/MS, moderately resistant/moderately susceptible; MS, moderately susceptible; S, susceptible*.

b *Cultivars had a different field reaction over several seasons*.

Chi-square analysis of the effect of cultivar of origin of the isolates on resistant and susceptible reactions was not significant in this study. Isolates that caused a susceptible or moderately susceptible reaction in SARDI tests on cv Nipper originated from a range of host cultivars *viz*: cvs Nipper, Cumra, PBA Flash, PBA Blitz, PBA Herald XT. Five isolates aggressive to cv Northfield were originally isolated from cvs Nipper, Northfield, Cumra and PBA Flash. Three other isolates that originated from cv Northfield caused small lesions (resistant or moderately resistant) on cvs Nipper and Northfield. As mentioned above a small amount of disease was occasionally recorded on cv Indianhead and ILL7537, including one isolate collected from cv Indianhead and three from ILL7537 in the stubble experiments. These isolates only developed a small amount of disease on the other hosts including cv Cumra. In the University of Melbourne tests the isolates derived from cv PBA Flash had a similar mean aggressive score to isolates from cv Nipper (means ± 95%CI: PBA Flash 4.62 ± 0.16; Nipper 4.45 ± 0.24). The two isolates from cv Northfield were ranked 8th and 26th out of 29 for aggressiveness while the single isolate from cv Aldinga was in the top 10 for aggressiveness (ranked 7th).

**Figure 2:**
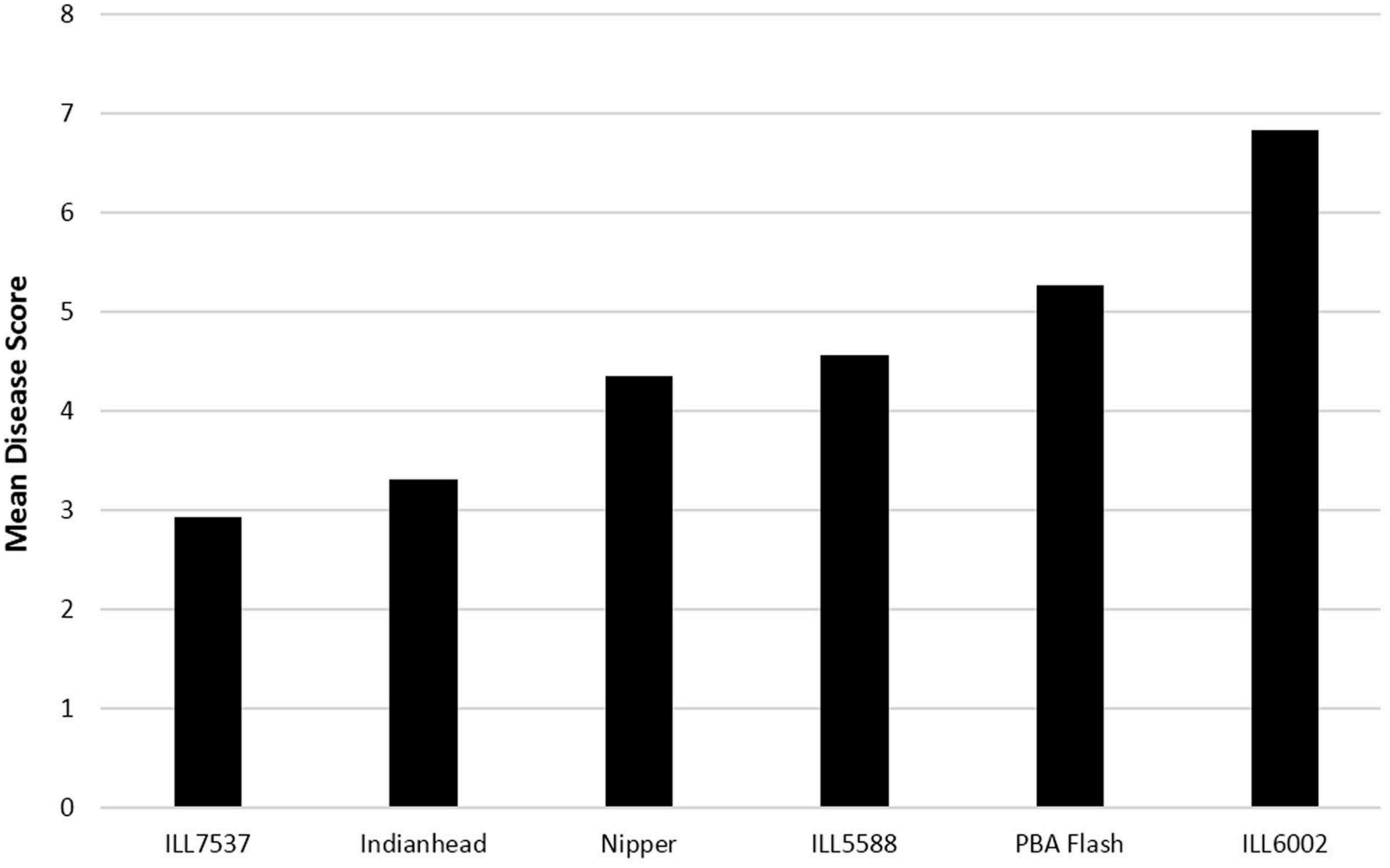
**Mean disease score for lentil accession at 28 dai with 29 *Ascochyta lentis* isolates**. Disease scored from 0 = no disease to 9 = severe disease/plant death. LSD 5% = 0.17. R, resistant; MR, moderately resistant; MR/MS, moderately resistant/moderately susceptible; MS, moderately susceptible; S, susceptible.

The disease scores of the 29 isolates screened on the six differentials at The University of Melbourne (Figure 2) overlapped, indicating a range of aggressiveness without distinction. Similar observations were made in the SARDI experiments (data not shown). Isolate/cultivar interaction scores showed that while ILL7537 was broadly resistant and ILL6002 susceptible to all isolates tested, significant differences in disease severity were produced by specific isolates on specific cultivars (Table 2). Similarly, eight isolates in the 2010–2013 SARDI collection caused a susceptible reaction on cvs Nipper and Northfield but the converse did not hold with an additional five isolates causing a susceptible reaction on cv Northfield. Only two isolates in the SARDI 2014 collection did not show the same reaction on cvs Nipper and Northfield, with one causing a moderately resistant reaction on cv Nipper but moderately susceptible on cv Northfield and vice versa for the other isolate. In The University of Melbourne tests, the cv PBA Flash produced the largest range of disease response (disease score range 3.75–6.74), followed in descending order by cvs Northfield, Nipper and Indianhead, thus providing data on specific isolate/cultivar combinations producing high, medium or low disease responses. Again similar observations were made in SARDI tests whereby the range of disease scores were highest in cv Northfield (0.1–26.2%APD), followed by Nipper (0.1–16.3%APD) and Indianhead (0–4.0%APD).

### Comparison of NVT lentil lines tested against *A. lentis* isolates in controlled conditions and in field trials

The field disease scores from Mallala 2013 and Horsham 2014 were more correlated with the disease scores resulting from the isolate aggressive on cv Nipper (isolate FT13013) than with the non-aggressive isolate FT10002 (Table 3). The correlation coefficient for comparison of isolate FT13013 with Mallala field scores = 0.82 (*P* < 0.001) and with Horsham = 0.87 (*P* < 0.001); the correlation coefficient for comparison of isolate FT10002 with Mallala = 0.53 (*P* < 0.02) and with Horsham (raw data) = 0.68 (*P* < 0.001). Results were comparable between the two field sites i.e., correlation coefficient = 0.92 (*P* < 0.001).

Five disease response groups were identified in this set of 27 lentil lines (Table 3). Group 1 only contained cv PBA Flash which was the most susceptible of the lines tested, and lacked field resistance. The breeding line CIPAL1421 was the only entry in Group 2 and was also susceptible to the isolates in the controlled testing but appeared to have some field resistance. Group 3 consisted of nine entries including cvs Nipper and Northfield that were susceptible to the isolate aggressive on cv Nipper (FT13013) but resistant to the control isolate Kewell. However two subgroups were identified that were susceptible or resistant to the recent isolate FT10002. Group 4 consisted of cvs PBA Blitz and PBA Giant which were moderately resistant to FT13013 but showed differential responses to isolate FT10002. Group 5 includes accessions that were resistant to the recent isolates (FT13013 and FT10002) but separated into two subgroups based on susceptibility or resistance to the control isolate Kewell.

## Discussion

The field experiments in this study were either designed as selection trials for plant breeders or were agronomic management trials and the disease assessment scales differed for these purposes. These trials were placed in fields in which lentil is part of the normal cropping rotation and management of the trials reflected local practices. Epidemics of ascochyta blight developed naturally and were similar to epidemics in surrounding commercial crops. Using isolates from breeding trials has the potential to bias results since there is a wide diversity of resistance genes in the trials, however additional isolates were collected from commercial fields. Irrespective of these issues the disease data presented here is sufficiently robust to confirm the change in field response of cv Nipper after 2010 which had also been observed in commercial crops. The phenotyping of *A. lentis* isolates on a differential host set was initiated independently in the two institutes and data were compared after the experiments were completed. While there were minor differences in the details of experimental procedures, the methodologies were very similar and the results from the two laboratories were in agreement. Although the results of growth room studies may not be directly translatable to the field, they do enable the effects of different environmental factors on disease occurrence and progress to be assessed and compared among host genotypes and growth stages. They also provide the necessary environmental controls and repeatability required for experiments to dissect the mechanisms of resistance in lentil specifically deployed against *A. lentis*, as well as the opportunity to select isolates and lentil cultivars with identified differential disease interactions. Despite the range of sources from which the isolates of *A. lentis* were collected, the phenotyping gave similar results between each source, demonstrating that a large percentage of isolates in the most recent collections were able to infect the previously resistant cv Nipper.

This study identified a natural diversity in aggressiveness of the *A. lentis* population leading to the loss of effective resistance in the widely used cv Nipper in lentil growing regions of southern Australia just 4 years after its commercialisation. A similar increase in aggressiveness of *A. lentis* isolates over a similar time period was detected in Canada, with possible breakdown of resistance in cv Laird (Ahmed et al., 1996; Banniza and Vandenberg, 2006). The correlation of isolate screening in controlled conditions with field observations indicates that isolates aggressive to cv Nipper have become more frequent and widespread in the *A. lentis* population, possibly as a selective response to the widespread presence of this cultivar in the farming system. This cultivar reached maximum cropping in 2012, covering 20% of the total lentil area, but was grown most frequently on the Yorke Peninsula in South Australia, comprising 30% of the lentil area (S. Crane, Seednet, personal communication). The aggressive isolates detected in southern Australia did not infect other resistant cultivars and breeding lines i.e. Indianhead, ILL7537 and PBA Herald XT.

Lentils are an important cash crop, especially on Yorke Peninsula where, anecdotally, the loss of resistance in cv Nipper was first observed. PBA cultivars with adapted traits are rapidly adopted in this region and cv Nipper was widely grown due to a premium price for its small round seed, its ability to withstand lodging and its resistance to both botrytis gray mold and ascochyta blight (Pulse Australia, 2011). Subsequently the area planted to cv Nipper has fallen and largely replaced by cv PBA Hurricane XT, made popular by its improved tolerance to Group B herbicides and resistance to ascochyta blight. This cultivar has now been planted over a greater area than cv Nipper. Commercialized in 2013 (Pulse Australia, 2013), in the following season 94% of South Australian PBA Hurricane XT seed sales went to Yorke Peninsula, while other regions had a wider spread of cultivar seed sales. It is predicted that this cultivar may occupy around 50% of the Australian lentil cropping area in the future (J. Sounness, PBSeeds Pty. Ltd., personal communication).

PBA Hurricane XT and a number of other cultivars, including PBA Ace, PBA Bolt and PBA Herald XT, share the parent line CDC Matador which in turn has Indianhead parentage. These cultivars maintain their resistant status in field conditions and are resistant to the isolates identified as aggressive on cv Nipper in the controlled screening. This indicates they contain different resistance gene(s) to cv Nipper although further research is required to confirm this. While no isolates in this study were able to completely overcome the resistances in cv Indianhead or ILL7537 some caused a moderately resistant reaction confirming an earlier study (Nguyen et al., 2001) in which isolates from Victoria were virulent on cv Indianhead. These results suggest a natural variability in the *A. lentis* population. Widespread planting of lentil cultivars with Indianhead/Matador heritage could lead to the selection of aggressive isolates against this resistance with a similar outcome to that observed on cv Nipper.

While the cvs Northfield and Nipper were field resistant to ascochyta blight in South Australia prior to 2006, it is apparent from this study that isolates able to infect them were already present in the *A. lentis* population, further evidence of a natural variability in the population. As well as the increased number of isolates that showed a susceptible reaction on cv Nipper, a significant number of the isolates from all collections were also able to infect cv Northfield mirroring results of Nasir and Bretag (1997a) in Victoria. This variability in the *A. lentis* population does not appear to be affected by the host cultivar although more studies are required to confirm this. A study specifically addressing host susceptibility and related isolate aggressiveness in wheat following epidemics of *Mycosphaerella graminicola* found that isolates recovered at the end of the season from moderately resistant cultivars were more aggressive than those from susceptible ones (Cowger and Mundt, 2002), and similar selective pressure on the *A. lentis* population may also be happening. Certainly in Canada, isolates collected in 1992 were found to be more aggressive than those collected in 1978 and 1985 (Ahmed et al., 1996). The cultivation in Australia of at least moderately resistant lentil cultivars indicates that continual monitoring of aggressiveness in the local *A. lentis* population is needed.

The cultivar of the naturally infested lentil stubbles had no influence on the number of lesions observed on adjacent lentil cultivars. However the proportion of aggressive isolates may increase with the introduction of resistant cultivars, as demonstrated by the controlled screening experiments whereby a greater number of isolates collected after 2010 were aggressive on cv Nipper. The isolates collected from stubble in 2013 and 2014 showed similar characteristics to isolates collected from the field in the same year, in that at least 50% were aggressive on cvs Nipper and Northfield. However there is no data for isolates produced from stubble prior to 2013, and so no information on how variability of isolates from stubble may have changed over the years of lentil cultivation in southern Australia. This study identified very low infection of *A. lentis* on cv PBA Hurricane XT in field trials and controlled experiments but the presence of low infection combined with the high selection pressure brought about by high cropping intensity could result in the selection of aggressive forms of the pathogen as seen with cv Nipper.

Ascochyta blight infection on lentil seed and pods can affect grain yield and quality through seed abortion and seed staining (Hawthorne et al., 2012). While infection on the foliage influences severity of seed and pod infection via rain-splash of conidia, cultivar responses to ascochyta blight on seed and foliage appear to differ (Hawthorne et al., 2012). In this study seed infection was low for cv Nipper in the field trials that were assessed, suggesting that seed resistance has remained effective. The genetics of resistance in cv Nipper are not understood however two recessive genes for foliar resistance have been identified in the parent line Indianhead (Ye et al., 2001) and a single recessive gene identified for seed resistance (Chowdhury et al., 2001). Two dominant genes have been identified in the other parent, cv Northfield, that confer resistance to foliar infection (Ford et al., 1999; Ye et al., 2001) but it is not known if these are the same genes that confer seed resistance (Tay and Slinkard, 1989; Chowdhury et al., 2001). Further research is required to understand the resistance in cv Nipper and whether shared or separate genes confer resistance to foliage and seed.

McDonald and Linde (2002) identified five evolutionary forces that contribute to the loss of effective resistance genes. Four of these five forces potentially demonstrate a high evolutionary risk for *A. lentis viz*. (1) large overseasoning populations survive on stubble maintaining virulent alleles, (2) asexual conidia are dispersed by air and the pathogen may transfer long distance on seed, (3) the reproduction system involves both annual sexual outcrossing and asexual propagules, and finally (4) the resistance genes are deployed in high cropping intensities. The fifth force is mutation rate but there is insufficient information in *A. lentis* to comment. McDonald and Linde (2002) hypothesized that pathogens like *A. lentis* that have mixed reproduction systems pose the highest risk of evolution since many new genotype combinations are created through recombination and these are “tested” in different environments, leading to the most fit types increasing in frequency through asexual reproduction. The rate of increase can be slowed by deploying genes in mixtures or in rotations through space and time which either reduces the efficiency or disrupts selection. They also state that these pathogens require most effort to achieve durable resistance and so breeding effort should concentrate on quantitative resistance which is renewed regularly to stay ahead of the pathogen. Consequently an ongoing study aimed at assessing temporal changes in aggressiveness of the *A. lentis* isolates on a range of elite Australian lentil cultivars is required to determine if potential selective evolution is occurring in relation to host resistances (Cowger and Mundt, 2002; Pariaud et al., 2009).

The identification of highly significant differences in disease reactions between specific isolates against specific cultivars in the phenotyping experiments provides opportunity for further study into the genetic differences involved. In particular, the broad range of disease severity from high to low among isolates on cvs PBA Flash, Northfield and Nipper will enable fine dissection of the interactions. The rapid loss of resistance in cv Nipper indicates there may be one or more major genes for resistance that have been rendered ineffective by changes in the pathogen population. However in addition to changes on specific hosts there is an apparent continuum of aggressiveness among the *A. lentis* isolates when assessing the mean reaction across the entire host set. This supports similar findings in the Canadian study (Ahmed et al., 1996), and is in broad agreement with the theory that the resistance that plants deploy against necrotrophs is polygenic, and can be quantitative as well as qualitative, rather than only the discreet responses seen against biotrophs (Thrall et al., 2005). While the genetic mechanisms of resistance that lentil uses against *A. lentis* are still poorly understood, reviews of this pathosystem report that both major and minor genes are inferred in the interaction, either singly or in complement, although the allelic nature of the genes is yet to be identified (Ye et al., 2001; Gupta et al., 2012). Races of necrotrophs have been identified in other pathosystems, for instance the *Phytophthora nicotianae*-tobacco interaction (Van Jaarsveld et al., 2002), based on the pathogen's ability to infect different cultivars expressing different resistance genes. However, the lack of cultivar specificity observed in the earlier Canadian study (Ahmed et al., 1996) indicates that the resistance mechanisms in lentil may be more complex. Recent evidence suggests that many plants respond to necrotrophs not only with quantitative responses but also with those activated depending on the pathogen species involved (Lai and Mengiste, 2013). Future planned sequencing of the transcriptome of lentil cultivars when challenged by *A. lentis* isolates with known aggressiveness will aid in uncovering these.

The strategy of the PBA lentil breeding program has been to develop lines with different sources of resistance to ascochyta blight from a range of parents, as demonstrated in Table 3. Many entries have the resistant cv CDC Matador in their pedigree while the resistance in cv PBA Jumbo2 has most likely come from parent CIPAL205, a line used extensively for ascochyta blight resistance in the Australian lentil breeding program. Relatively minor resistances have also been pyramided and one of the resulting cultivars (Boomer) shows effective resistance in the field and also in controlled conditions although the origin of this resistance is unclear since neither of the parent lines, cvs Digger and Palouse, are resistant to *A. lentis* (Ford et al., 1999; Sambasivam, 2011). However the agronomic success of individual lines such as cv Nipper and now cv PBA Hurricane XT has led to the rapid and dominant uptake of single cultivars. This intensity threatens the durability of ascochyta blight resistance in PBA Hurricane XT and related lines, and if resistance is rendered ineffective this will reduce the number of resistant sources that can be used in the Australian lentil breeding program. Better genetic understanding and molecular tools for rapid inclusion of major and minor genes is paramount to maintaining resistance to ascochyta blight in the Australian lentil industry. While additional sources of resistance must be sought, it is also important to encourage cultural practices that maintain disease resistance.

In conclusion, a broad range of aggressiveness and natural variability exists among recent Australian isolates of *A. lentis*. Also significant differences in disease severity exist among specific isolates, enabling researchers' choice of highly aggressive isolates for targeted resistance breeding efforts and individual isolate/cultivar combinations with high, medium and low levels of disease severity for future investigation of the potentially differential defense responses. Detailed understanding of the genetics of resistance to *A. lentis* is essential for the successful future deployment of ascochyta blight resistance in lentil cultivars.

## Author contributions

LM and MR conducted field experiments; JD, LM, and MR assessed and analyzed data from field trials; GS and RF conducted and analyzed controlled experiments at Melbourne University; MHR, MK, and JD conducted controlled experiments and stubble experiments at SARDI; JD analyzed data from SARDI experiments; JD, GS, and RF wrote the manuscript; all authors reviewed the manuscript before submission.

### Conflict of interest statement

The authors declare that the research was conducted in the absence of any commercial or financial relationships that could be construed as a potential conflict of interest.
